# Dopamine-Functionalized Gold Nanoparticles for Colorimetric Detection of Histamine

**DOI:** 10.1021/acsomega.3c10123

**Published:** 2024-04-02

**Authors:** Romnick B. Unabia, Renzo Luis D. Reazo, Rolen Brian P. Rivera, Melbagrace A. Lapening, Jahor L. Omping, Ryan M. Lumod, Archie G. Ruda, Noel Lito B. Sayson, Gerard Dumancas, Roberto M. Malaluan, Arnold A. Lubguban, Gaudencio C. Petalcorin, Rey Y. Capangpangan, Felmer S. Latayada, Arnold C. Alguno

**Affiliations:** † Research Center on Energy Efficient Materials (RCEEM), Premier Research Institute in Science and Mathematics (PRISM), Mindanao State University − Iligan Institute of Technology, A. Bonifacio Avenue, Iligan City 9200, Philippines; ‡ Department of Chemistry, Loyola Science Center, The University of Scranton, Scranton, Pennsylvania 18510, United States; § Center for Sustainable Polymers, MSU-Iligan Institute of Technology, Iligan City 9200, Philippines; ∥ Department of Mathematics and Statistics, Mindanao State University-Iligan Institute of Technology, Iligan City 9200, Philippines; ⊥ Mindanao State University at Naawan Campus, Naawan Misamis Oriental 9023, Philippines; # Caraga State University-Main Campus, Ampayon, Butuan City 8600, Philippines

## Abstract

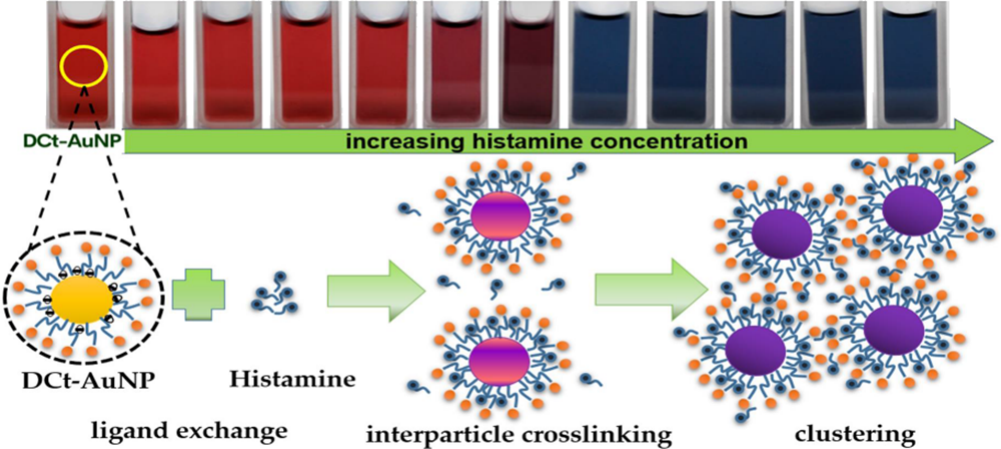

Histamine, a primary biogenic amine (BA) generated through the decarboxylation of amino acids, concentration increases in protein-rich foods during deterioration. Thus, its detection plays a crucial role in ensuring food safety and quality. This study introduces an innovative approach involving the direct integration of dopamine onto gold nanoparticles (DCt-AuNP), aiming at rapid histamine colorimetric detection. Transmission electron microscopy revealed the aggregation of uniformly distributed spherical DCt-AuNPs with 12.02 ± 2.53 nm sizes upon the addition of histamine to DCt-AuNP solution. The Fourier-transform infrared (FTIR) spectra demonstrated the disappearance of the dicarboxy acetone peak at 1710 cm^–1^ along with the formation of well-defined peaks at 1585 cm^–1^, and 1396 cm^–1^ associated with the N–H bending modes and the aromatic C=C bond stretching vibration in histamine molecule, respectively, confirming the ligand exchange and interactions of histamine on the surface of DCt-AuNPs. The UV–vis spectra of the DCt-AuNP solution exhibited a red shift and a reduction in surface plasmon resonance (SPR) peak intensity at 518 nm along with the emergence of the 650 nm peak, signifying aggregation DCt-AuNPs with increasing histamine concentration. Notably, color transitions from wine-red to deep blue were observed in the DCt-AuNP solution in response to histamine, providing a reliable colorimetric signal. Dynamic Light Scattering (DLS) characterization showed a significant increase in the hydrodynamic diameter, from ∼15 to ∼1690 nm, confirming the interparticle cross-linking of DCt-AuNPs in the presence of histamine. This newly developed DCt-AuNP sensor provides colorimetric results in less than a minute that exhibits a remarkable naked-eye histamine detection threshold of 1.57 μM and a calculated detection limit of 0.426 μM, making it a promising tool for the rapid and sensitive detection of histamine.

## Introduction

Ensuring food safety and quality has been one of the current challenges today due to an increasing number of food poisoning incidents according to the World Health Organization (WHO) with an estimated 600 million illnesses per year leading to 420,000 deaths annually.^1,2^ Foodborne diseases are related to unhygienic food handling, processing, and inadequate temperature storage conditions.^2,3^ The Food and Drug Administration (FDA) and European Food Safety Authority (EFSA) highlighted that consumption of biogenic amine-contaminated foods can lead to adverse health effects, including allergic reactions, migraines, and even life-threatening conditions at high concentrations.^3,4^ Biogenic amines (BAs) such as histamine, putrescine, and cadaverine are nitrogenous molecules generated through the decarboxylation of amino acids in protein-rich foods during the extended storage time of food products.^5,6^ In this regard, BAs are considered to be a freshness and quality indicator as their concentration increases with food spoilage.^6,7^

Numerous methodologies have been implemented for qualitative and quantitative assessment of BAs, including chromatographic methods,^8^ electrochemistry,^9^ enzyme-linked assays,^10^ and capillary electrophoresis.^11^ While these techniques have been proven reliable, they often need to improve on certain constraints such as time-consuming sample preparation, tedious procedures, and the requirement of specialized expensive equipment, making them unsuitable for on-site investigation.^12^ Thus, there is a need for an innovative alternative approach that can provide rapid and cost-effective detection of BAs.

Over the past decade, gold nanoparticles (AuNPs) have been considered a promising candidate in drug delivery, bioimaging, and biosensing applications owing to their excellent biocompatibility, simple synthesis, size-tunable surface plasmon resonance (SPR), and versatile surface chemistry for ease in functionalization.^13−15^ Owing to its unique optical properties, AuNPs have been employed as biosensors, giving colorimetric output during dispersion and aggregation activity.^16−18^ Related studies on biogenic amine detection using unmodified/bare AuNPs exhibited a strong cross-reactive response with various biogenic amines such as spermine, spermidine, tryptamine histamine, and melamine.^18−22^ Accordingly, unmodified AuNPs are highly unstable and easily triggered by any chemical stimuli, leading to nonspecific analyte interaction because of their high surface-to-volume ratio limiting their practical application.^16,17^

To address this problem, nanoparticles are usually coated by an organic capping layer to provide a protective coating that could enhance their selectivity with its target analyte. Mercapto (−SH) and amino (−NH_2_) functional groups are commonly used for capping AuNP because of their high affinity to gold.^17,23,24^ Exploring the functionalization of AuNPs with dopamine, a biomolecule with amine (−NH_2_) and a catechol group (C_6_H_6_O_2_), holds tremendous potential in enhancing its sensitivity and selectivity for BA detection, as it demonstrates fast polymerization in the presence of organic amines, which is ideal for biosensors. Aside from that, dopamine contributes to the stability and antifouling ability of AuNP through covalent backfilling, providing a steric hindrance against aggregation.^25−27^ Furthermore, a colorimetric detection method for BAs based on dopamine polymerization in the presence of BAs on the surface of AuNPs has been reported.^27^ However, the above studies carried out three-way processes: (1) reduction of AuNP, (2) stabilization with thiolated-polyethylene glycol (PEG-SH), and (3) addition of dopamine solution on the PEG-SH functionalized AuNPs to enhance its sensitivity, making the synthesis process time-consuming and costly. Furthermore, the colorimetric detection of BAs in their study takes 4 h of incubation of BA in dopamine-added PEG-SH functionalized AuNPs.

In light of the aforementioned research gap, this study aims to investigate the novel approach of direct dopamine functionalization of citrate-reduced AuNPs (Ct-AuNP) to be used as a colorimetric detection system. This study utilized a histamine analyte as a representative biogenic amine to validate the feasibility of the direct dopamine-functionalized AuNPs (DCt-AuNPs) for rapid colorimetric detection. Transmission electron microscopy (TEM) was employed to determine the structural modification and colloidal properties of the AuNPs upon dopamine functionalization and the addition of histamine. To investigate the surface chemistry and molecular interactions occurring on the nanoparticle’s surface during functionalization and histamine testing, Fourier transform infrared (FTIR) was used. To evaluate the attenuation of the SPR properties of DCt-AuNPs upon the addition of histamine and the changes in the hydrodynamic diameter sizes and size distribution of DCt-AuNPs, ultraviolet–visible (UV–vis) spectroscopy, and dynamic light scattering (DLS) measurements were utilized, respectively. A proposed mechanism for the interaction of histamine molecules with DCt-AuNP’s surface was also presented.

## Experimental Section

Materials: Gold(III) chloride hydrate (HAuCl_4_) (99.995% trace metals basis), trisodium citrate dihydrate (Na_3_Ct) (ACS reagent, ≥ 99.0%), dopamine hydrochloride (H8502), and histamine (Analytical Standard, 97%) were acquired from Sigma-Aldrich. Unless otherwise stated, all precursors used were analytical grade and used without additional purifications. Milli-Q ultrapure water (18.2 MΩ·cm) (Merck KGaA, Germany) was used as a solvent for all experiments.

Synthesis of DCt-AuNPs: 50 mL of 3.0 molar ratio (MR) Ct-AuNPs was prepared by combining 34.0 mM Na_3_Ct and 0.5 mM HAuCl_4_ solutions. The HAuCl_4_ solution was placed in a water bath and heated to 95 °C with vigorous stirring. Once the desired temperature was reached, an appropriate amount of Na_3_Ct was added rapidly to the HAuCl_4_ solution, causing a gradual color shift from yellow to wine-red. The synthesis of Ct-AuNPs was considered complete when the solution’s color stabilized, typically within ∼5 min. Subsequently, the solution was left to cool to room temperature.

After the solution had cooled, 50 mL of the as-prepared Ct-AuNP solution was stirred at 360 rpm and added with 0.250 mL of 1 mM dopamine hydrochloride solution to synthesize DCt-AuNPs. The mixture was stirred continuously for a duration of 4 h at room temperature. The resulting solution was subsequently stored at 4 °C for further use.

Characterization of DCt-AuNPs: The structural and colloidal properties of the synthesized AuNPs were studied using TEM. Micrographs of the AuNPs were acquired using a JEM 2100 Plus LaB_6_ (JEOL, Japan) TEM equipped with STEM capabilities with resolution up to 0.14 nm at 200 kV. An ample amount of the AuNP colloidal solution was dropped on the 3 mm Cu grid with Formvar/carbon supporting film using Pasteur pipettor and dried for 15 min. The samples’ Cu grid was mounted on a TEM holder and inserted into a dry pump multistation for 24 h for outgassing before characterization.

The particle size and size distribution of each sample were determined through image analysis using ImageJ downloaded (https://imagej.net/ij/). The TEM image was converted to an 8-bit type and calibrated using the image scale bar to obtain the pixel/nm value. The “bandpass filter” image processing tool at appropriate pixel values was used, and the adjusting the “threshold” tool to highlight the individual particle. The data used to calculate the particle size and distribution was obtained using the “analyzed particle” tool by adjusting the circularity and size of the particle of interest.

FTIR was employed to identify the characteristic bonds associated with dopamine-functionalized AuNPs and the presence of histamine. Infrared transmittance spectra were recorded using a Shimadzu IR-TRACER100 (Shimadzu, Japan) within the 4000–400 cm^–1^ spectral range, with 60 scans. In an Eppendorf tube, 2.0 mL of AuNP colloidal solution was centrifuged at 9000 rpm for 30 min. After removing the supernatant, a small amount of the precipitate was placed on the FTIR sample platform and allowed to air-dry before analysis.

UV–vis spectroscopy was used to determine the surface plasmon resonance of the synthesized solution. UV–vis spectra were acquired using a Thermo-Scientific GENESYS 10S spectrometer (Thermo-Scientific, Massachusetts, US) with a 1.8 nm spectral bandwidth. The analysis was carried out in the 200–1000 nm range with 1.0 nm resolution.

DLS measurements were carried out to determine the hydrodynamic diameter sizes of the colloidal solutions, utilizing the Nanotrac Wave II Analyzer (Microtrac, Inc., Pennsylvania, USA). A 1 mL portion of the synthesized AuNP solution was loaded in the sample cell holder. To evaluate hydrodynamic diameter sizes, “distribution analysis” was set in the Microtrac Flex 11 software for 60 s. All measurements were taken under room temperature conditions (25 °C).

Colorimetric Test with a Histamine analyte: The analyte solutions were prepared by dissolving varying amounts of histamine precursor in ultrapure water to attain concentrations ranging from 1 to 100 ppm. Subsequently, 100 μL of the histamine solution was added to a 2.0 mL DCt-AuNP solution for colorimetric testing. The resulting solution was then photographed to monitor any observable colorimetric responses as the histamine concentration increased. Additionally, UV–vis spectroscopy was employed to assess the changes in the SPR peaks of AuNPs following the addition of analytes. It is worth noting that all analyte solutions were freshly prepared and all experiments were conducted under ambient room temperature conditions.

Selectivity test on the DCt-AuNP solution: To assess the colorimetric responses of the DCt-AuNP with various analytes such as organic solvents, weak acids, and different biological substances containing amino groups, i.e., putrescine, cadaverine, inosine, and histamine, available in the laboratory, selectivity analyses were conducted. A 2.0 mL of DCt-AuNP colloidal solution was placed in a cuvette and then 100 μL analyte solutions were added. The mixture was swirled for 1 min and allowed to react for 5 min. Attenuation of the SPR peak and other spectral changes were observed by using UV–vis spectroscopy.

Time-dependent stability test on the DCt-AuNP solution: For practical application, the DCt-AuNP solutions should demonstrate colloidal stability through negligible attenuation of the SPR peak and no color transitions of the solution during extended storage durations. Hence, a time-dependent stability test of DCt-AuNP colloidal solutions was conducted. Here, DCt-AuNP solutions were stored at 4 °C for various durations, i.e., 0, 1, 3, 7, 14, and 21 days, and then 2 mL aliquots were taken for analysis using UV–vis spectroscopy to evaluate the SPR peak with increasing storage time.

## Results and Discussion

**Figure 1:**
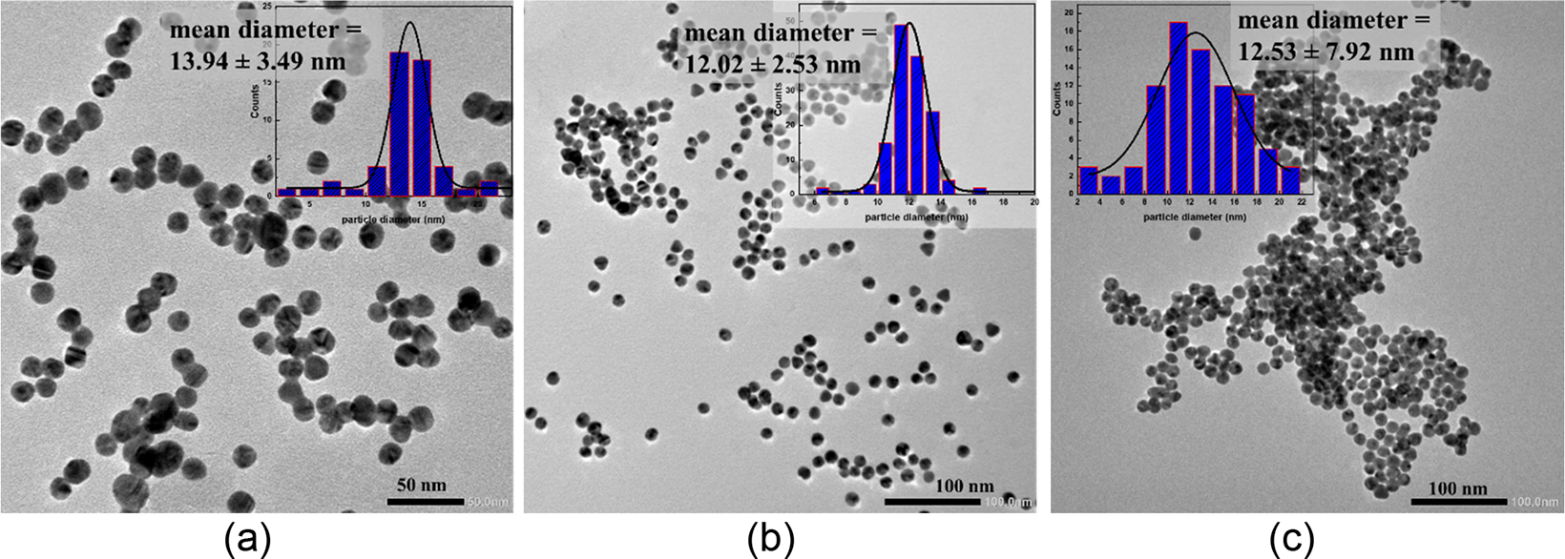
TEM images show the dispersion, particle size, and size distribution of (a) Ct-AuNPs, (b) DCt-AuNP, and (c) DCt-AuNP with histamine; The inset is the graph of its particle diameter histogram.

The colorimetric detection system presented in this study was prepared via direct dopamine functionalization on Ct-AuNPs.^13,27^ The TEM images shown in Figure 1 provide significant insights into the structural and colloidal properties of synthesized AuNPs. The bare Ct-AuNP depicts spherical nanoparticles having an average size of 13.94 ± 3.49 nm with good size uniformity, as depicted in the histogram in Figure 1a. Upon dopamine functionalization, DCt-AuNPs exhibit uniform spherical nanoparticles with a 12.02 ± 2.53 nm average size as shown in Figure 1b. It can be observed that direct dopamine functionalization does not greatly affect the average particle size of the AuNPs but rather allows precise tuning of surface properties as observed in the homogeneity in the dispersion, highlighting the effective prevention of aggregation of dopamine ligands. Comparatively, the well-dispersed spherical nanoparticles of DCt-AuNPs entail potential advantages for sensing applications due to their size uniformity and nanometer-scale sizes.

Standard histamine testing was conducted to validate the feasibility of the DCt-AuNP sensor for rapid colorimetric detection. Histamine was used as a model BA since it has been linked to allergic inflammatory responses and notable food poisoning outbreaks upon consumption of spoiled foods.^2^ TEM image of DCt-AuNP solution in the presence of histamine in Figure 1c shows the spherical morphology with an average particle size of 12.53 ± 7.92 nm analogous to bare DCt-AuNPs but highlights the aggregation of AuNPs. The wide distribution of the particle sizes of DCt-AuNPs could be associated with the clustering of particles upon the addition of histamine.

**Figure 2:**
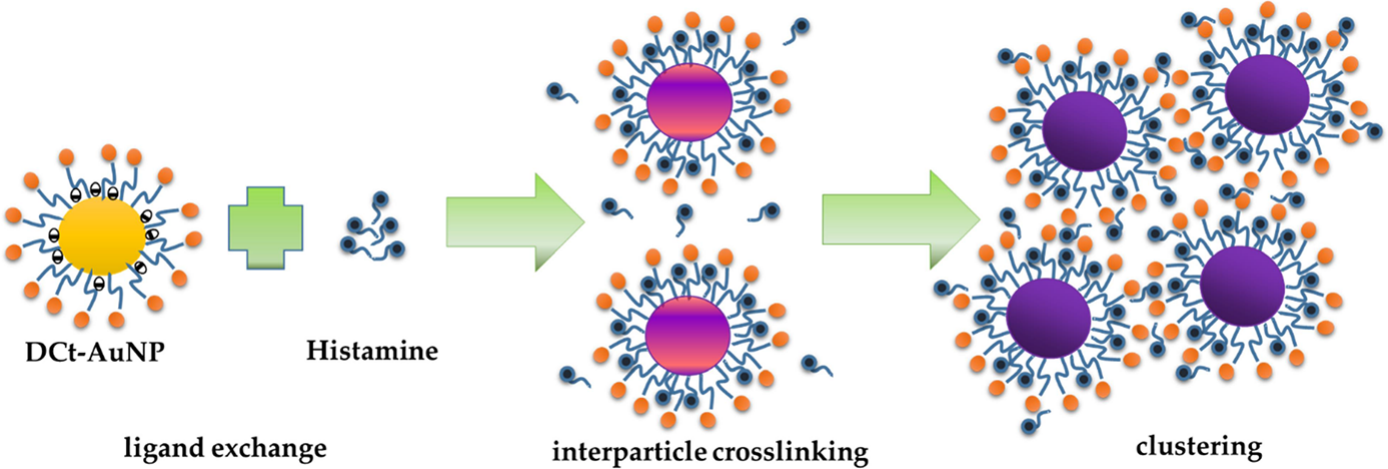
Proposed mechanism shows the ligand exchange between citrates and histamine molecules and the histamine role as a bidentate linker for DCt-AuNPs leading to interparticle cross-linking upon the addition of histamine to DCt-AuNPs.

A proposed mechanism for the interaction between histamine and DCt-AuNPs was presented in this study, as illustrated in Figure 2. The imidazole ring in histamine could coordinate with the gold atoms on the nanoparticle surface, displacing the dicarboxy acetone through ligand exchange. The adsorption of histamine on the surface of AuNPs resulted in the modification of the surface chemistry, which could induce changes in the SPR of DCt-AuNPs.^27^ On the other hand, the presence of excess histamine molecules in the solution could lead to an aggregation mechanism through interparticle cross-linking wherein histamine plays as a bidentate linker for neighboring DCt-AuNPs through the aliphatic amino groups and imidazole ring, which present as an interaction site for neighboring particles resulting to aggregation of AuNPs.^19,25,28^

**Figure 3:**
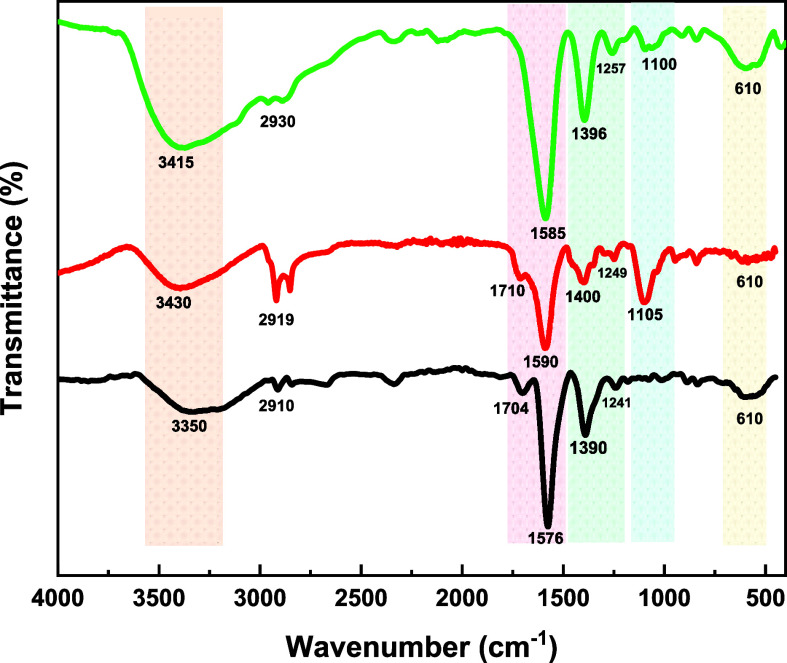
FTIR spectra of AuNP solution showing the OH stretching at ∼3400 cm^–1^, the C–H stretching of organic components at ∼2900 cm^–1^, −C(=O)– bonds at ∼1700 cm^–1^, C=O stretching at ∼1500 cm^–1^, the C–H stretching of amine groups at ∼1400 cm^–1^, the C–N stretching at ∼1250 cm^–1^, the C–O–C/C–C stretching at 1100 cm^–1^, and metal–ligand vibrations at 610 cm^–1^ present in (black dashed line) Ct-AuNP, (red dashed line) DCt-AuNP, and (green dashed line) DCt-AuNP with 100 ppm histamine.

To provide valuable insights into the surface chemistry and molecular interactions occurring on the nanoparticle’s surface during functionalization processes and histamine testing, FTIR spectroscopy was utilized. Figure 3 shows the FTIR spectra for all of the prepared colloidal samples. The strong peaks observed at 1390 and 1576 cm^–1^ were associated with the −COO^–^ stretching and carboxylate groups (C=O stretching) of citrate molecules, respectively. In addition, the peak at 1704 cm^–1^ is indicative of the presence of ketonic carbon–oxygen bonds confirming the formation of dicarboxy acetone which capped the surface of AuNPs.^28,29^

Upon dopamine functionalization, peaks around 1576 cm^–1^, and 1704 cm^–1^ red-shift to a higher wavenumber to 1590 cm^–1^, and 1710 cm^–1^ attributed to the amine stretching of dopamine, i.e., corroborating the functionalization of −NH_2_ group onto the surface of AuNPs.^30,31^ Moreover, the shifting of the peaks at 1241 and 1390 cm^–1^ to 1249 and 1400 cm^–1^, associated with the C–N stretching and C–H bending vibrations, respectively, along with the appearance of a peak at 1105 cm^–1^ for C–O–C/C–C stretching, further validates the successful functionalization of dopamine onto the surface of AuNPs.^30,31^

Additionally, the peaks around 2900–2800 and 3500–3200 cm^–1^ were ascribed to the C–H stretching of the organic components and the −OH stretching of the hydroxyl groups of citrate molecules during the reduction of gold precursor as well as to the presence of aliphatic carbon–hydrogen bonds and the hydrogen bonding or coordination interactions of dopamine molecules with AuNPs during functionalization. Subsequently, the ketonic carbon–oxygen bond at 1710 cm^–1^ was still observed even after functionalization, indicating the concurrence of dopamine and dicarboxy acetone capping the surface of AuNPs, as illustrated in Figure 3.^29^

Upon histamine testing, the ligand exchange proposed mechanism can be supported by the disappearance of the peak around 1710 cm^–1^ attributed to the ketonic carbon–oxygen bonds of dicarboxy acetone along with the prominence of the 1257 cm^–1^ peak associated with the C–N stretching vibrations of histamine’s amino (NH_2_) groups and enhanced 610 cm^–1^ peak associated with metal–ligand vibrations between AuNPs and organic ligands. The ligand exchange was possible since −NH of histamine has a higher affinity with Au than −OH, as shown in Figure 3.^23,24^

Accordingly, the observed well-defined peaks at 1585 and 1396 cm^–1^ are associated with the N–H bending modes and the aromatic C=C bond stretching vibration in histamine molecule, which further confirms the interaction of histamine with DCt-AuNPs through its amine (−NH) groups. Consequently, a gradual increase in peak intensity at ∼3500 cm^–1^, ascribed to aromatic −NH and −OH stretching vibrations of DCt-AuNP in the presence of histamine, was observed, suggesting an enhanced interaction between dopamine and histamine molecules through hydrogen bonding, i.e., associated with the clustering of DCt-AuNPs.^29^

**Figure 4:**
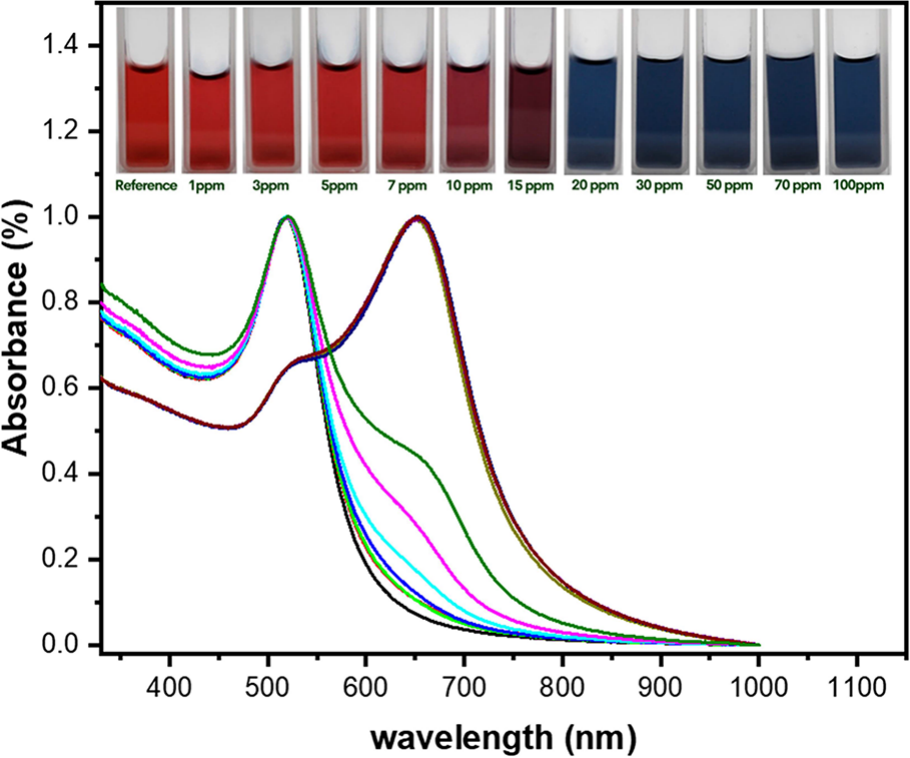
UV–vis absorbance spectra of DCt-AuNP added with increasing histamine concentration showing the reduction of the SPR peak of AuNPs at 518 nm along with the emergence of an additional peak at 650 nm. (black dashed line) bare DCt-AuNP and DCt-AuNP with histamine content of (red dashed line) 1 ppm, (green dashed line) 3 ppm, (blue dashed line) 5 ppm, (turquoise dashed line) 7 ppm, (pink dashed line) 10 ppm, (olive dashed line) 15 ppm, (yellow dashed line) 20 ppm, (gray dashed line) 30 ppm, (indigo dashed line) 50 ppm, (brown dashed line) 70 ppm, and (black dashed line) 100 ppm. The inset displays the colorimetric response of the actual DCt-AuNP solutions tested with the histamine analyte.

In the same way, the aggregation or clustering of nanoparticles facilitated by histamine concentration could further modify the SPR peak of AuNPs, as shown in Figure 4. The UV–vis spectral data portrayed a noticeable red shift coupled with an attenuated SPR intensity at 518 nm along with the emergence of a new ∼650 nm peak upon increasing histamine concentration which relates to the formation of clustered nanoparticles. This observation is in agreement with the proposed mechanism that histamine molecules could possibly adsorb onto AuNP surfaces and induce interparticle cross-linking between AuNPs. It should be taken into account that the swelling around the 650 nm spectral region was already evident at 1 ppm histamine concentration, indicating our colorimetric sensor’s sensitivity within an expedited time frame of <1 min. However, a saturation of aggregation behavior was observed at 20 ppm concentration as no further significant change in the SPR peak.

On the other hand, the aggregation of AuNPs dramatically affects the SPR property which alters the possible frequencies of light that can be absorbed or scattered, resulting in a color change observed by the naked eye. Noteworthy colorimetric assessments through visual inspection manifested a pronounced hue transition in the DCt-AuNP ensemble from wine-red to deep blue with increasing histamine concentration, as observed from the inset of Figure 4. Distinct color transitions observable with the naked eye can be detected at 7 ppm histamine concentration, i.e., 1.54 μM calculated concentration of histamine in the colloidal solutions.

The attenuation of the SPR peak at 518 nm and the emergence of the 650 nm peak signal the aggregation of AuNPs upon interaction with histamine. To quantify the degree of aggregation of DCt-AuNPs in the presence of histamine analyte, we determine the aggregation index (AI), i.e., the ratio between the absorbance of the peak at 650 nm versus the absorbance of the peak at 518 nm.^27,29^ Mathematically, the aggregation index (AI) is expressed as
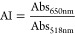
1

**Figure 5:**
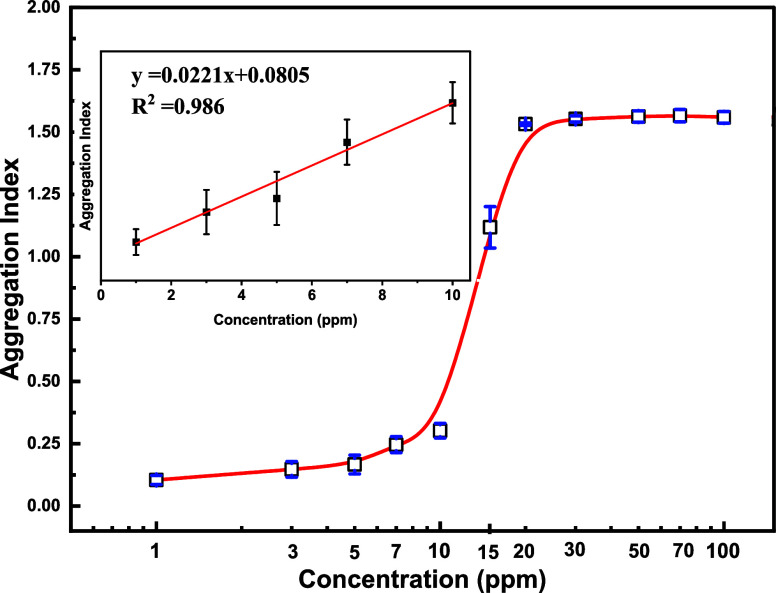
Ratio between the absorbance of the peak at 650 nm and the SPR peak at 518 nm indicates the aggregation index (AI), i.e., quantification of the degree of aggregation of DCt-AuNP upon the addition of histamine with increasing concentration. Inset: The calibration curve of histamine detection at 1–10 ppm range. The error bars represent the standard deviation of the mean of the three independent experiments.

Figure 5 shows the AI values of three independent repetitions for DCt-AuNP with an increasing histamine concentration. In the absence of the histamine, the measured AI has a value of 0.066 ± 0.018. Upon the addition of histamine in the DCt-AuNP solution, the AI value increases, reaching a value of 1.56 ± 0.027, which correlates to the increasing hydrodynamic measurements of DCt-AuNP solution in the presence of histamine, suggesting a meaningful estimation of DCt-AuNP aggregation. Higher concentrations of histamine exhibit a saturation value of the AI at 20 ppm.

**Figure 6:**
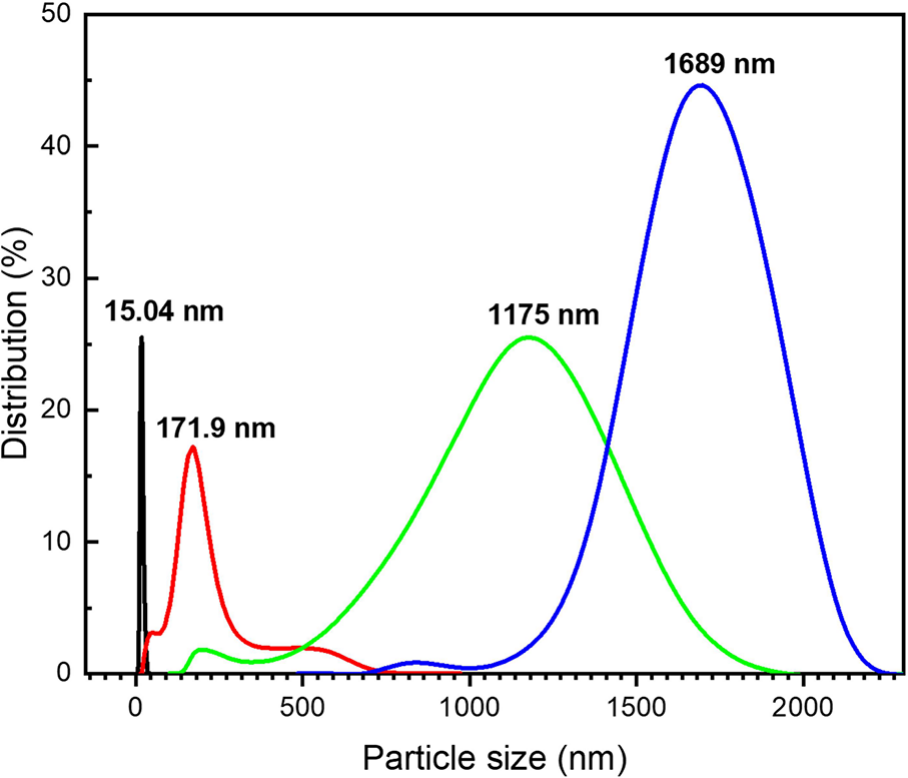
DLS measurements showing colloidal properties of (black dashed line) DCt-AuNP solution added with (red dashed line) 10 ppm, (green dashed line) 50 ppm, and (blue dashed line) 100 ppm histamine, i.e., hydrodynamic diameter sizes and distribution.

The presence of histamine on the nanoparticle surface could alter the overall surface charge and electrostatic interactions, which, in turn, might affect the colloidal stability of the nanoparticles and their interactions with other molecules or surfaces, as observed in the DLS measurement in Figure 6. Increasing the concentration of histamine added to the DCt-AuNP solution, a drastic increase in the hydrodynamic diameter of nanoparticles and its distribution from 15.04 to 1689 nm was observed, which confirms the clustering or aggregation of DCt-AuNPs. This aggregation can be attributed to the electrostatic interactions between the amino and imidazole functional groups of histamine with the hydroxyl and amine groups of dopamine on the nanoparticle surface, leading to the interparticle cross-linking of AuNPs into clusters (see also Figure 2).

**Figure 7:**
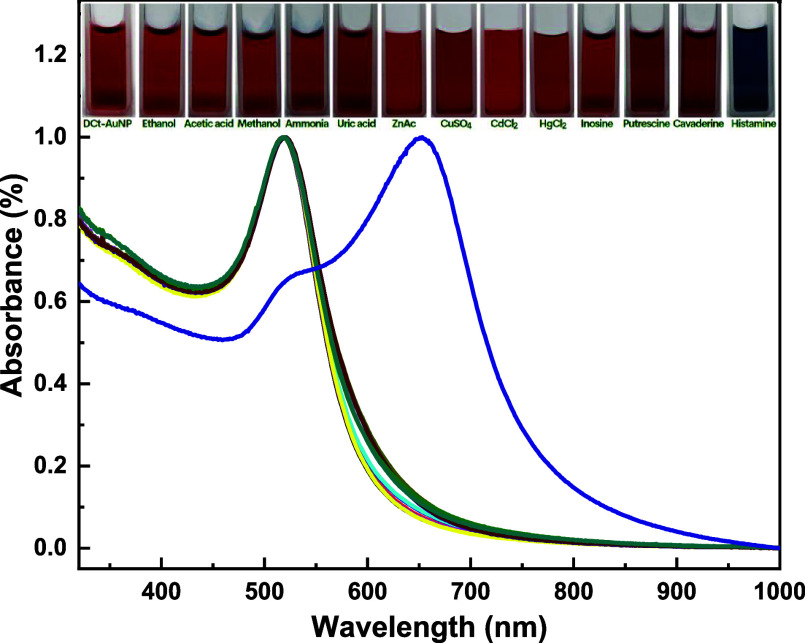
UV–vis spectra show the absorbance of (black dashed line) DCt-AuNP solution tested with various analytes such as (red dashed line) EtOH −100 ppm, (green dashed line) AcOH −100 ppm, (blue dashed line) MeOH −100 ppm, (turquoise dashed line) NH_3_ −100 ppm, (pink dashed line) UA −100 ppm, (yellow dashed line) Ino −100 ppm, (brown dashed line) ZnAc −100 ppm, (purple dashed line) CuSO_4_-100 ppm, (indigo dashed line) CdCl_2_ −100 ppm, (olive dashed line) HgCl_2_ −100 ppm, (dark green dashed line) Put-100 ppm, (teal dashed line) Cad-100 ppm, and (purple dashed line) His-100 ppm for selectivity assessment. The inset shows the actual appearance of the DCt-AuNP solution tested with various analytes at 100 ppm.

To further assess the colorimetric responses of the DCt-AuNP sensor, we conducted a selectivity test with the organic solvents, weak acids, and other BAs, i.e., related to the spoilage process of all proteinaceous foods, available in the laboratory at various concentrations (10, 50, and 100 ppm).^34^Figure 7 displays the UV–vis spectra of DCt-AuNP solution with 100 ppm of different analyte solutions, i.e., ethanol (EtOH), acetic acid (AcOH), methanol (MeOH), ammonia (NH_3_), uric acid (UA), inosine (Ino), zinc acetate (ZnAc), copper sulfate (CuSO_4_), cadmium chloride (CdCl_2_), mercury chloride (HgCl_2_), putrescine (Put), cadaverine (Cad), and histamine (His). The UV–vis characterization reveals no significant attenuation with the surface plasmon resonance peak of DCt-AuNP in the presence of various analytes at increasing concentrations from 10 to 100 ppm except for the histamine analyte. In addition, we also tested our colloidal solution with other BAs such as putrescine and cadaverine, and inorganic compounds (ZnAc, CuSO_4_, CdCl_2_, HgCl_2_) at 100 ppm, but still no substantial alterations with the SPR peak except the swelling of the baseline around 650 nm. This implies that it would take higher concentrations of putrescine, cadaverine, and tested inorganic compounds to cause changes in the SPR property of our DCt-AuNP sensor. The inset of Figure 7 shows the actual images of DCt-AuNP solutions displaying the distinct color transitions for histamine compared to the other analyte solution from wine-red to deep blue hue. These variations of the optical properties of DCt-AuNP solution in the presence of histamine analyte signify the selective response of our direct dopamine-functionalized AuNPs to histamine. Even though toxic effects were attributed to the presence of several BAs, histamine was considered to have detrimental effects on human health when consumed at higher concentrations.^35,36^

Lastly, the DCt-AuNP colorimetric sensor’s performance was systematically evaluated by comparing it with existing methods with respect to linear dynamic range, sensing time, and limit of detection (LOD) values with various biogenic amines. Previously, Lapenna et al.^19^ reported a synthesis of “naked” AuNPs via laser ablation in liquid for histamine detection. A linear range of 0.2–0.4 μM histamine concentration with a detection limit of 0.2 μM was observed after 10 min reaction time. Abbasi-Moayed et al.^20^ obtained unmodified AuNPs through the Turkevich method which reported a multiplex detection of spermine, spermidine, histamine, and tryptamine having a linear concentration range of 0.1–10.0 μM with a limit of detection around 0.2 μM for histamine after 10 min reaction time. In addition, Li et al.,^23^ developed a sensor probe using thiolated polyethylene glycol (PEG-SH) functionalized AuNPs with the addition of dopamine that exhibits strong responses to histamine, putrescine, cadaverine, spermine, spermidine, tyramine, and tryptamine. A linear dynamic range of 1–100 μg/mL was reported with a limit of detection at 2.8 μg/mL for histamine with an incubation time of 4 h.

Accordingly, our method on DCt-AuNPs as a colorimetric sensor for histamine reported a linear relationship at a histamine concentration range of 1–10 ppm with a regression equation of *y* = 0.0221*x* + 0.0.0805 having a correlation coefficient of 0.986 (see inset B of Figure 5). Notably, our colorimetric sensor demonstrated a straightforward process with swift response, enhanced sensitivity, and highly selective detection to histamine having a detection limit of 0.426 μM at an expedited time frame of <1 min, outperforming the majority of previously reported methods, as detailed in Table 1.

**Table 1:** Comparison on Analyte Responses, Linear Dynamic Range, Sensing Time, and Limit of Detection Value for the Detection of Various Biogenic Amines between Different Literatures

sensor probe	analyte detected	concentration	linear range	sensing time (min)	detection limit	references
naked AuNPs^a^	histamine	0–1 μM	0.2–0.4 μM	10	0.2 μM	Lapenna et al.^19^
unmodified citrate-reduced AuNPs^b^	spermine, spermidine, tryptamine histamine	0.1–10.0 μM	0.1–10.0 μM	10	0.3 μM	Abbasi-Moayed et al.^20^
bare citrate-reduced AuNPs^b^	histamine	0.6–18 μM	0.6–18 μM	15	0.6 μM	El- Nour et al.^21^
citrate-stabilized AuNPs^b^	melamine	0.1–2 mg/L	0.1–2 mg/L	15	0.5 mg/L	Kumar et al.^22^
citrate-reduced gold nanoparticle^b^ functionalized with thiolated polyethylene glycol (PEG-SH) and addition of dopamine	histamine, putrescine, cadaverine, spermine, spermidine, tyramine, and tryptamine	1–100 μg/mL	1–100 μg/mL	4 h	2.8 μg/mL	Li et al.^23^
dopamine-functionalized AuNPs	histamine	1–100 ppm	1–10 ppm	<1	0.426 μM	our work

a Via laser ablation in liquid.

b Via Turkevich Method.

**Figure 8:**
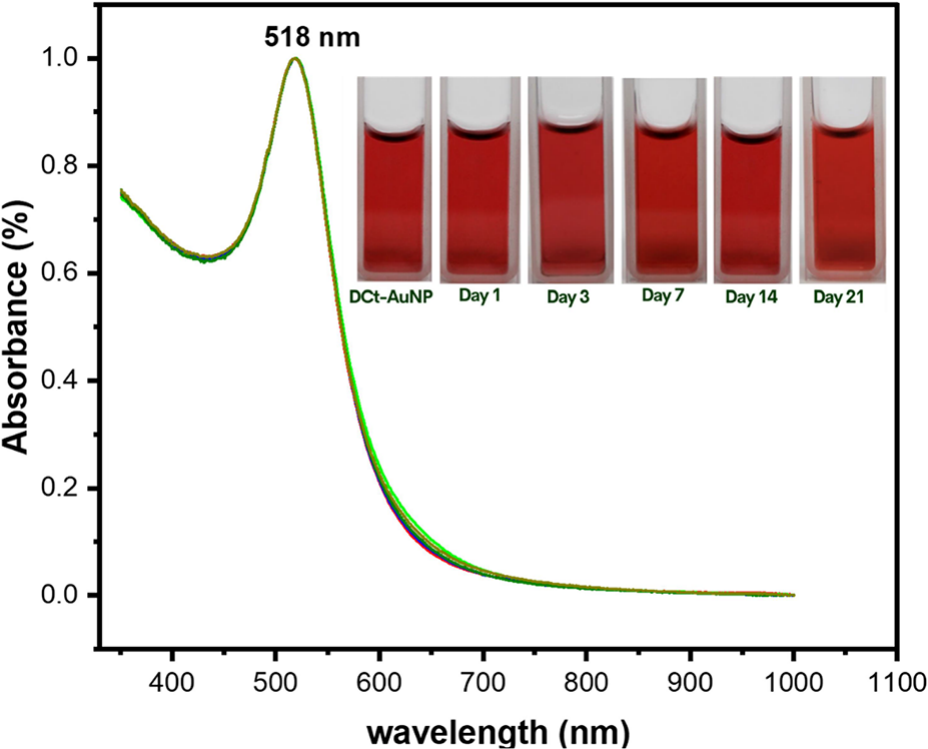
Stability test showing the absorbance of the SPR peak of DCt-AuNP solution stored at 4 °C at various durations, i.e., (black dashed line) as-synthesized, (red dashed line) 1 day, (green dashed line) 3 days, (blue dashed line) 7 days, (dark green dashed line) 14 days, and (olive dashed line) 21 days. The inset displays the actual appearance of the DCt-AuNP solutions.

We conducted time-dependent stability to demonstrate the colloidal stability of the DCt-AuNP solution for practical application evaluated through attenuation of the SPR peak and color transitions of the solution during extended storage durations. Figure 8 shows the UV–vis spectra of DCt-AuNP stored at 4 °C for various durations. It can be observed from the graph that there are no distinct changes in the SPR peak of AuNPs with an increasing storage time, indicating that the surface plasmonic core remains unchanged. In addition, the color of the DCt-AuNP solutions remained wine-red even after 21 days of storage at 4 °C, indicating the colloidal stability of the solution (see the inset of Figure 8). The amine groups of dopamine observed in the 1635 cm^–1^ peak of the FTIR analysis may undergo chemisorption onto the gold surface through Au–N coordination bonds resulting in the formation of a monolayer or a self-assembled dopamine layer on the nanoparticles’ surface (as illustrated in Figure 2) which then offers steric repulsion between particles that contributes homogeneity and colloidal stability of nanoparticles.^32,33^ Hence, the DCt-AuNP sensor was stable enough for a long storage duration for histamine detection.

## Conclusions

This study successfully reported a novel approach to synthesizing dopamine-functionalized gold nanoparticles (DCt-AuNPs) and colorimetric detection histamine. The introduction of dopamine in the functionalization of gold nanoparticles facilitated the precise tuning of surface properties which inhibits aggregation of colloidal solution over a long storage duration (21 days). Moreover, the developed colorimetric sensor demonstrated a rapid (within an expedited time frame of <1 min), sensitive, and highly selective detection with histamine among various organic solvents, weak acids, and other BAs related to the spoilage process of proteinaceous foods tested, i.e., inosine, putrescine, and cadaverine. Colorimetric assessments demonstrated a noticeable hue transition in the DCt-AuNP solution from wine-red to deep blue upon adding histamine analyte. A linear correlation (*R*^2^ = 0.986) was obtained between the aggregation index of DCt-AuNP absorbance and histamine concentration range at 1–10 ppm having a detection limit of 0.426 μM. Future research endeavors will investigate further the assessment and monitoring of real meat freshness using DCt-AuNP colorimetric sensors. Henceforth, the DCt-AuNP detection system is promising for the in-field application of visualization and colorimetric detection of histamine in food products without the aid of highly specialized equipment.
